# High-density lipoprotein cholesterol levels and prognosis in non-ischemic dilated cardiomyopathy

**DOI:** 10.3389/fendo.2026.1843944

**Published:** 2026-06-26

**Authors:** Chao Gong, Mengmeng Zhou, Shenzhen Gong, Chunxiao Liu, Jinying Zhang

**Affiliations:** 1 Department of Cardiology, The First Affiliated Hospital of Zhengzhou University, Zhengzhou, China; 2 Department of Anaesthesiology, West China Hospital, Sichuan University, Chengdu, China; 3 Department of Clinical Laboratory, The Eighth People’s Hospital of Zhengzhou, Zhengzhou, China

**Keywords:** cardiac magnetic resonance, dilated cardiomyopathy, high-density lipoprotein cholesterol, prognosis, pulmonary hypertension

## Abstract

**Background:**

Although low high-density lipoprotein cholesterol (HDL-C) is associated with poor outcomes in heart failure, its specific prognostic role in non-ischemic dilated cardiomyopathy (DCM) remains undefined. This study aimed to investigate the association between HDL-C levels and long-term clinical outcomes in patients with DCM.

**Methods:**

We retrospectively enrolled 297 patients with DCM who underwent cardiac magnetic resonance between January 2017 and October 2024. The primary endpoint was a composite of all-cause mortality and heart transplantation; the secondary endpoint additionally included heart failure readmission. The associations between HDL-C and clinical outcomes were assessed using Kaplan-Meier analysis, Cox regression, restricted cubic spline (RCS), and sensitivity analyses.

**Results:**

During a median follow-up of 35 months, 41 (13.8%) and 97 (32.7%) patients experienced the primary and secondary endpoints, respectively. Kaplan-Meier analysis demonstrated that patients with higher HDL-C had significantly better event-free survival for both the primary and secondary endpoints (log-rank P < 0.001 for both). In multivariable Cox regression models, lower HDL-C was consistently and independently associated with an increased risk of the primary (hazard ratio [HR] 0.19, 95% confidence interval [CI]: 0.05–0.68, P = 0.011) and secondary (HR 0.15, 95% CI: 0.07–0.34, P < 0.001) endpoints. After further adjustment for echocardiographically estimated pulmonary artery systolic pressure, the effect estimate for the association between HDL−C and clinical outcomes was attenuated (primary endpoint: HR 0.31, 95% CI: 0.08–1.11, P = 0.072; secondary endpoint: HR 0.19, 95% CI: 0.08–0.44, P < 0.001). RCS curves showed a linear relationship between HDL-C and both endpoints (P for nonlinearity > 0.05 for both). Sensitivity analyses confirmed the robustness of the results.

**Conclusions:**

Lower HDL-C levels were independently associated with adverse outcomes in patients with non-ischemic DCM, an association partly attenuated by adjusting for pulmonary hypertension markers. As a readily available prognostic biomarker, HDL-C may inform risk stratification in this population.

## 1 Introduction

Non-ischemic dilated cardiomyopathy (DCM) is a leading cause of heart failure, defined by left ventricular or biventricular dilation and systolic dysfunction without significant coronary artery disease or abnormal loading conditions (1, 2). Despite advances in pharmacotherapy, device interventions, and transplantation, patients with DCM remain at substantial risk of mortality, driven predominantly by sudden cardiac death and progressive heart failure (3). However, due to the high clinical heterogeneity of DCM, conventional risk stratification tools often lack sufficient accuracy for predicting long-term outcomes. Therefore, there is an urgent need to identify new, feasible biomarkers to improve prognostic assessment in this population.

In recent years, dyslipidemia has been increasingly recognized as a critical factor influencing cardiovascular outcomes (4). High-density lipoprotein cholesterol (HDL-C) has long been considered to possess multiple cardioprotective properties, including anti-atherosclerotic, anti-inflammatory, antioxidant, and anti-apoptotic effects (5). Numerous studies have reported that low HDL-C levels are associated with adverse cardiovascular events in patients with coronary artery disease and ischemic heart failure (6–9). Some research also suggests that HDL-C may influence myocardial remodeling and heart failure progression through non-traditional mechanisms, such as immune modulation and vascular function maintenance (10, 11). However, whether HDL-C retains independent prognostic value specifically in patients with non-ischemic DCM remains unclear. Systematic evidence in this distinct etiologic subgroup is lacking. This knowledge gap is noteworthy. The pathophysiology of non-ischemic DCM differs substantially from ischemic heart disease, and findings from other heart failure populations may not be directly applicable.

Pulmonary hypertension (PH) is a common complication in DCM, primarily resulting from chronically elevated pulmonary venous pressure secondary to left-sided heart failure (12). The presence of PH not only increases right ventricular workload but also correlates strongly with adverse outcomes (13). Emerging studies have suggested a close relationship between HDL-C and pulmonary vascular function, with reduced HDL-C potentially promoting pulmonary vasoconstriction or endothelial dysfunction, thereby contributing to the development or exacerbation of PH (14–16). These findings raise the possibility that the association between HDL-C and prognosis in DCM might be partially explained by PH.

Therefore, the present study had two objectives. The first was to evaluate the independent prognostic value of HDL-C levels in patients with non-ischemic DCM. The second was to investigate descriptively whether the association between HDL-C and adverse clinical outcomes is attenuated after adjustment for PH, thereby examining whether PH may partially account for this relationship. Collectively, these findings may provide new insights for risk stratification in this population and inform future hypothesis-driven research.

## 2 Methods

### 2.1 Study population

This retrospective cohort study consecutively enrolled patients diagnosed with DCM at the First Affiliated Hospital of Zhengzhou University from January 2017 to October 2024. Inclusion criteria were based on impaired left ventricular ejection fraction (LVEF < 50%) and elevated left ventricular end-diastolic dimension (LVEDD ≥ 55 mm) on cardiac magnetic resonance (CMR). Patients were excluded if they had any of the following: (1) ischemic heart disease, defined as a history of myocardial infarction or coronary revascularization, > 50% luminal stenosis in any of the three major coronary arteries on coronary angiography or computed tomography, or ischemic pattern of late gadolinium enhancement (LGE) on CMR; (2) moderate or severe primary mitral or aortic valve diseases, hypertensive heart disease, acute myocarditis, chronic excessive alcohol consumption, or other types of cardiomyopathy, including hypertrophic, restrictive, infiltrative, peripartum, chemotherapy-induced, or tachycardia-induced cardiomyopathy; (3) poor image quality on CMR, or refusal to provide informed consent. This study was approved by the Ethics Committee of the First Affiliated Hospital of Zhengzhou University (2025-KY-1581-001) and conducted in accordance with the Declaration of Helsinki. Written informed consent was obtained from all participants.

### 2.2 Data collection

Baseline data were extracted from electronic medical records. Demographics included age, sex, body mass index (BMI), systolic blood pressure (SBP), diastolic blood pressure (DBP), and heart rate (HR). Clinical characteristics and comorbidities comprised smoking and drinking status, New York Heart Association class (NYHA II-IV), hypertension, diabetes mellitus, atrial fibrillation (AF), and left bundle branch block (LBBB). Medication use included angiotensin receptor–neprilysin inhibitors (ARNI), angiotensin-converting enzyme inhibitors (ACEI), angiotensin receptor blockers (ARB), β-blockers, mineralocorticoid receptor antagonists (MRAs), diuretics, and digoxin. Device therapy (implantable cardioverter-defibrillator [ICD] and cardiac resynchronization therapy [CRT]) was documented as present or absent. BMI was calculated as weight (kg) divided by height squared (m^2^).

Fasting blood samples were collected from all participants at admission. Our central clinical laboratory quantified the following measurements using standardized protocols: leukocyte count, albumin, N-terminal pro–B-type natriuretic peptide (NT-proBNP), troponin T (TnT), serum creatinine, estimated glomerular filtration rate (eGFR), fasting plasma glucose (FBG), glycated hemoglobin (HbA1c), total cholesterol (TC), triglycerides (TG), HDL-C, and low-density lipoprotein cholesterol (LDL-C).

CMR was performed on a 3.0T scanner (Skyra, Siemens Healthineers, Erlangen, Germany), and all measurements were analyzed by qualified cardiovascular imaging specialists. Structural parameters included interventricular septal (IVS) thickness, left ventricular posterior wall (LVPW) thickness, right atrial anterior-posterior diameter (RAAPD), right ventricular end-diastolic diameter (RVEDD), left atrial anterior-posterior diameter (LAAPD), and left ventricular end-diastolic diameter (LVEDD). Functional and volumetric parameters comprised left ventricular end-diastolic volume (LVEDV), end-systolic volume (LVESV), ejection fraction (LVEF), and left ventricular mass (LVmass). These parameters were indexed to body surface area (BSA).

Transthoracic echocardiography was performed by experienced technicians. Key parameters included mitral inflow E and A wave peak velocities (pulsed-wave Doppler), tricuspid regurgitation (TR) velocity (continuous-wave Doppler), and left ventricular fractional shortening (LVFS). Estimated pulmonary artery systolic pressure (ePASP) was calculated using the modified Bernoulli equation: ePASP = 4 × (TR velocity)^2^ + estimated right atrial pressure, where right atrial pressure was estimated from inferior vena cava diameter and respiratory variation. Echocardiographic LVEF (LVEF-echo) was determined using the biplane Simpson method.

### 2.3 Follow-up and clinical endpoints

Patients were followed up until May 31, 2025, through outpatient clinic visits, electronic medical record review, and telephone interviews. The primary endpoint was a composite of all-cause mortality and heart transplantation. The secondary endpoint included the primary endpoint components plus heart failure readmission. Time-to-event was calculated from the date of the baseline CMR examination to the occurrence of the first clinical endpoint. Patients who experienced no endpoint event were censored at the date of their last documented follow-up.

### 2.4 Statistical analysis

All statistical analyses were conducted using R version 4.4.0 (R Foundation for Statistical Computing, Vienna, Austria) and GraphPad Prism version 10.2.0 for Macintosh (GraphPad Software, San Diego, California, USA). A two-tailed P value < 0.05 was considered statistically significant. Continuous variables were expressed as mean ± standard deviation (SD) or median (interquartile range, IQR), depending on the distribution assessed by the Shapiro-Wilk test. Categorical variables were presented as counts and percentages. Between-group comparisons were performed using Student’s t-test or Mann-Whitney U test for continuous variables and chi-square or Fisher’s exact test for categorical variables, as appropriate.

To assess the associations between HDL-C levels and clinical outcomes, receiver operating characteristic (ROC) curve analysis was used to determine optimal cut-off values. Survival curves were established using the Kaplan–Meier method and compared using the log-rank test. Univariate and multivariate Cox proportional hazards regression models were applied to estimate hazard ratios (HRs) with 95% confidence intervals (CIs). The models were built sequentially: Model 1 was unadjusted; Model 2 was adjusted for the most clinically relevant and well-established confounders, including age, sex, BMI, NYHA class, use of standard heart failure medications (ARNI/ACEI/ARB, β-blockers, and MRAs), log NT-proBNP, and LVEF; and Model 3 further included ePASP. ePASP was chosen as the primary echocardiographic measure of PH because it integrates TR velocity and right atrial pressure, providing a more direct estimate of systolic pulmonary artery pressure.

Restricted cubic spline (RCS) regression was applied to examine the potential nonlinear relationship between HDL-C and the primary and secondary endpoints. Nonlinearity was tested using the likelihood ratio test. To assess the robustness of our findings, we performed several sensitivity analyses based on Model 2: (1) applying Firth’s penalized likelihood Cox regression; (2) further adjusting for the inflammatory marker leukocyte count and the nutritional marker albumin; and (3) replacing ePASP with TR velocity in Model 3. Spearman correlation analysis was used to evaluate the relationship between HDL-C and echocardiographic markers of PH (ePASP and TR velocity).

## 3 Results

### 3.1 Baseline clinical characteristics and imaging parameters

The overall study population comprised 297 patients with a median age of 50 years; 73% were male. During a median follow-up of 35 months (interquartile range: 17–54 months), 41 participants (13.8%) reached the primary endpoint, which consisted of all-cause mortality (36 patients, 12.1%) and heart transplantation (6 patients, 2.0%). One patient died from pulmonary infection within 8 months after heart transplantation. The secondary endpoint occurred in 97 patients (32.7%), of whom 56 (18.9%) experienced heart failure readmission without reaching the primary endpoint. As shown in **Table 1**, patients who reached the primary endpoint (n = 41) differed significantly from event-free patients (n = 256) across multiple clinical and laboratory parameters. Specifically, the event group had lower BMI, DBP, HDL-C, and TC levels, as well as higher NT-proBNP and TnT concentrations. They also exhibited a higher prevalence of AF and less frequent use of ARNI/ACEI/ARB or β-blockers (all P < 0.05). CMR findings revealed reduced IVS and LVPW thickness, together with increases in RAAPD, RVEDD, LVEDD, LVEDVI, and LVESVI. Both CMR-derived LVEF and echocardiographic LVEF (LVEF-echo) were decreased, and LVFS was also reduced. In addition, TR velocity, ePASP, and E peak were elevated, and the time to event occurrence was shorter (all P < 0.05).

**Table 1:** Baseline clinical characteristics and imaging parameters.

Variables	Total (n = 297)	Without primary endpoint (n = 256)	With primary endpoint (n = 41)	P-value	Without secondary endpoint (n = 200)	With secondary endpoint (n = 97)	P-value
Age, years	50 ± 15	49 ± 14	52 ± 19	0.291	49 ± 14	52 ± 17	0.151
Males, n (%)	216 (73)	189 (74)	27 (66)	0.287	151 (76)	65 (67)	0.123
BMI, kg/m^2^	26.0 ± 5.5	26.4 ± 5.5	23.5 ± 5.1	<0.001	26.4 ± 5.4	25.3 ± 5.7	0.072
SBP, mmHg	123 ± 19	124 ± 18	118 ± 20	0.070	125 ± 19	120 ± 19	0.032
DBP, mmHg	78 ± 15	80 ± 15	72 ± 14	0.002	80 ± 15	75 ± 14	0.006
Heart rate, bpm	86 ± 17	86 ± 17	83 ± 18	0.303	86 ± 18	84 ± 17	0.303
Current and former smoker, n (%)	116 (39)	104 (41)	12 (29)	0.166	83 (42)	33 (34)	0.215
Current and former drinker, n (%)	92 (31)	82 (32)	10 (24)	0.326	68 (34)	24 (25)	0.106
NYHA class, n (%)				0.852			0.689
II	125 (42)	109 (43)	16 (39)		82 (41)	43 (44)	
III	102 (34)	88 (34)	14 (34)		72 (36)	30 (31)	
IV	70 (24)	59 (23)	11 (27)		46 (23)	24 (25)	
Comorbidity, n (%)							
Hypertension	86 (29)	77 (30)	9 (22)	0.287	58 (29)	28 (29)	0.981
Diabetes	50 (17)	40 (16)	10 (24)	0.164	28 (14)	22 (23)	0.061
AF	34 (11)	24 (9.4)	10 (24)	0.014	16 (8.0)	18 (19)	0.007
LBBB	39 (13)	30 (12)	9 (22)	0.072	20 (10)	19 (20)	0.022
Laboratory examination							
Leukocyte count, ×10^9^/L	7.27 ± 2.91	7.10 ± 2.48	8.28 ± 4.73	0.048	7.16 ± 2.61	7.48 ± 3.46	0.271
Albumin, g/L	39.6 ± 5.1	39.8 ± 5.2	38.8 ± 4.8	0.229	39.8 ± 5.4	39.3 ± 4.4	0.264
Log NT-proBNP, pg/mL	3.25 ± 0.61	3.20 ± 0.61	3.60 ± 0.50	<0.001	3.17 ± 0.63	3.43 ± 0.54	<0.001
TnT, ug/L	0.02 [0.01, 0.03]	0.02 [0.01, 0.03]	0.02 [0.01, 0.05]	0.006	0.02 [0.01, 0.03]	0.02 [0.01, 0.05]	0.026
Creatinine, umol/L	106 ± 135	108 ± 144	94 ± 64	0.598	114 ± 161	91 ± 52	0.987
eGFR, mL/min/1.73m^2^	86 ± 25	87 ± 25	82 ± 25	0.054	87 ± 25	85 ± 25	0.330
TC, mmol/L	3.88 ± 1.11	3.93 ± 1.12	3.59 ± 1.06	0.028	4.07 ± 1.14	3.51 ± 0.95	<0.001
TG, mmol/L	1.46 ± 1.12	1.49 ± 1.16	1.32 ± 0.80	0.607	1.53 ± 1.25	1.32 ± 0.80	0.115
HDL-C, mmol/L	1.00 ± 0.30	1.01 ± 0.29	0.89 ± 0.29	0.013	1.04 ± 0.29	0.90 ± 0.28	<0.001
LDL-C, mmol/L	2.39 ± 0.90	2.40 ± 0.91	2.31 ± 0.80	0.454	2.48 ± 0.95	2.21 ± 0.75	0.017
FBG, mmol/L	5.34 ± 1.58	5.30 ± 1.55	5.59 ± 1.73	0.241	5.31 ± 1.65	5.40 ± 1.43	0.117
HbA1C, %	6.25 ± 1.16	6.22 ± 1.16	6.40 ± 1.13	0.245	6.17 ± 1.13	6.39 ± 1.19	0.174
Medications, n (%)							
ARNI/ACEI/ARB	256 (86)	227 (89)	29 (71)	0.002	177 (89)	79 (81)	0.098
β-blockers	237 (80)	212 (83)	25 (61)	0.001	163 (82)	74 (76)	0.294
MRAs	242 (81)	212 (83)	30 (73)	0.140	162 (81)	80 (82)	0.759
Diuretics	216 (73)	186 (73)	30 (73)	0.945	138 (69)	78 (80)	0.038
Digoxin	85 (29)	70 (27)	15 (37)	0.224	56 (28)	29 (30)	0.734
Devices, n (%)							
ICD	11 (3.7)	10 (3.9)	1 (2.4)	>0.999	7 (3.5)	4 (4.1)	0.753
CRT	9 (3.0)	8 (3.1)	1 (2.4)	>0.999	6 (3.0)	3 (3.1)	>0.999
CMR parameters							
IVS thickness, mm	9.49 ± 1.45	9.57 ± 1.46	8.94 ± 1.28	0.010	9.64 ± 1.40	9.17 ± 1.51	0.007
LVPW thickness, mm	9.50 ± 1.39	9.58 ± 1.36	8.98 ± 1.46	0.011	9.62 ± 1.35	9.24 ± 1.44	0.022
RAAPD, mm	39 ± 9	38 ± 8	43 ± 12	0.003	38 ± 9	40 ± 10	0.054
RVEDD, mm	18.9 ± 4.7	18.7 ± 4.4	20.5 ± 5.8	0.048	18.6 ± 4.6	19.5 ± 4.9	0.135
LAAPD, mm	40 ± 9	39 ± 8	42 ± 12	0.057	38 ± 8	42 ± 10	<0.001
LVEDD, mm	69 ± 8	69 ± 8	72 ± 10	0.023	68 ± 8	71 ± 9	0.002
LVEDVI, mL/m^2^	148 ± 54	143 ± 49	179 ± 71	0.005	143 ± 49	159 ± 62	0.053
LVESVI, mL/m^2^	114 ± 49	110 ± 45	144 ± 66	0.003	109 ± 44	125 ± 57	0.044
LVEF, %	24 ± 9	25 ± 9	21 ± 8	0.013	25 ± 9	23 ± 8	0.140
LVmassi, g/m^2^	92 ± 32	92 ± 31	97 ± 35	0.619	92 ± 31	93 ± 34	0.977
Echo parameters							
E peak, m/s	0.79 ± 0.28	0.78 ± 0.27	0.92 ± 0.32	0.006	0.77 ± 0.26	0.86 ± 0.30	0.024
A peak, m/s	0.66 ± 0.27	0.66 ± 0.26	0.66 ± 0.32	0.652	0.67 ± 0.27	0.65 ± 0.27	0.754
TR velocity, m/s	2.51 ± 0.52	2.45 ± 0.48	2.91 ± 0.61	<0.001	2.43 ± 0.48	2.67 ± 0.56	<0.001
ePASP, mmHg	34 ± 13	33 ± 12	45 ± 15	<0.001	32 ± 12	39 ± 14	<0.001
LVEF-echo, %	35 ± 9	35 ± 9	30 ± 8	<0.001	36 ± 10	32 ± 9	0.030
LVFS, %	17.1 ± 5.5	17.6 ± 5.5	14.6 ± 4.4	0.002	17.5 ± 5.5	16.4 ± 5.5	0.120
Primary endpoint time, months	38 ± 24	39 ± 24	29 ± 23	0.011	36 ± 22	43 ± 27	0.028
Secondary endpoint time, months	31 ± 23	33 ± 23	19 ± 19	<0.001	36 ± 22	21 ± 21	<0.001

Data are presented as mean ± SD, counts (percentages), or median [interquartile range], as appropriate.

BMI, body mass index; SBP, systolic blood pressure; DBP, diastolic blood pressure; NYHA, New York Heart Association; AF, atrial fibrillation; LBBB, left bundle branch block; NT-proBNP, N-terminal pro-B-type natriuretic peptide; TnT, Troponin T; eGFR, estimated glomerular filtration rate; TC, total cholesterol; TG, total triglyceride; HDL-C, high-density lipoprotein cholesterol; LDL-C, low-density lipoprotein cholesterol; ARNI, angiotensin receptor neprilysin inhibitor; ACEI, angiotensin-converting enzyme inhibitor; ARB, angiotensin receptor blocker; MRAs, mineralocorticoid receptor antagonists; SGLT2i, sodium glucose co-transporter 2 inhibitors; ICD, implantable cardioverter-defibrillators; CRT, cardiac resynchronization therapy; IVS, interventricular septum; LVPW, left ventricular posterior wall; RAAPD, right artrial anterior posterior diameter; RVEDD, right ventricular end-diastolic diameter; LAAPD, left atrial anterior posterior diameter; LVEDD, left ventricular end-diastolic diameter; LVEDVi, left ventricular end-diastolic volume index; LVESVi, left ventricular end-systolic volume index; LVEF, left ventricular ejection fraction; LVmassi, left ventricular mass index; TR, tricuspid regurgitation; ePASP, estimated pulmonary artery systolic pressure; LVFS, left ventricular fractional shortening.

In the secondary endpoint analysis, patients with events (n = 97) had lower SBP, DBP, HDL-C, TC, and LDL-C, as well as higher NT-proBNP and TnT concentrations, compared to event-free patients (n = 200). They also showed a greater prevalence of AF and LBBB and more frequent use of diuretics (all P < 0.05). CMR revealed reduced thickness of the IVS and LVPW, together with increases in LAAPD, LVEDD, and LVESVI. Echocardiographic LVEF was decreased, while E peak, TR velocity, and ePASP were elevated. Moreover, the time to event was significantly shorter in the event group (all P < 0.05).

### 3.2 Survival analysis

**Figure 1:**
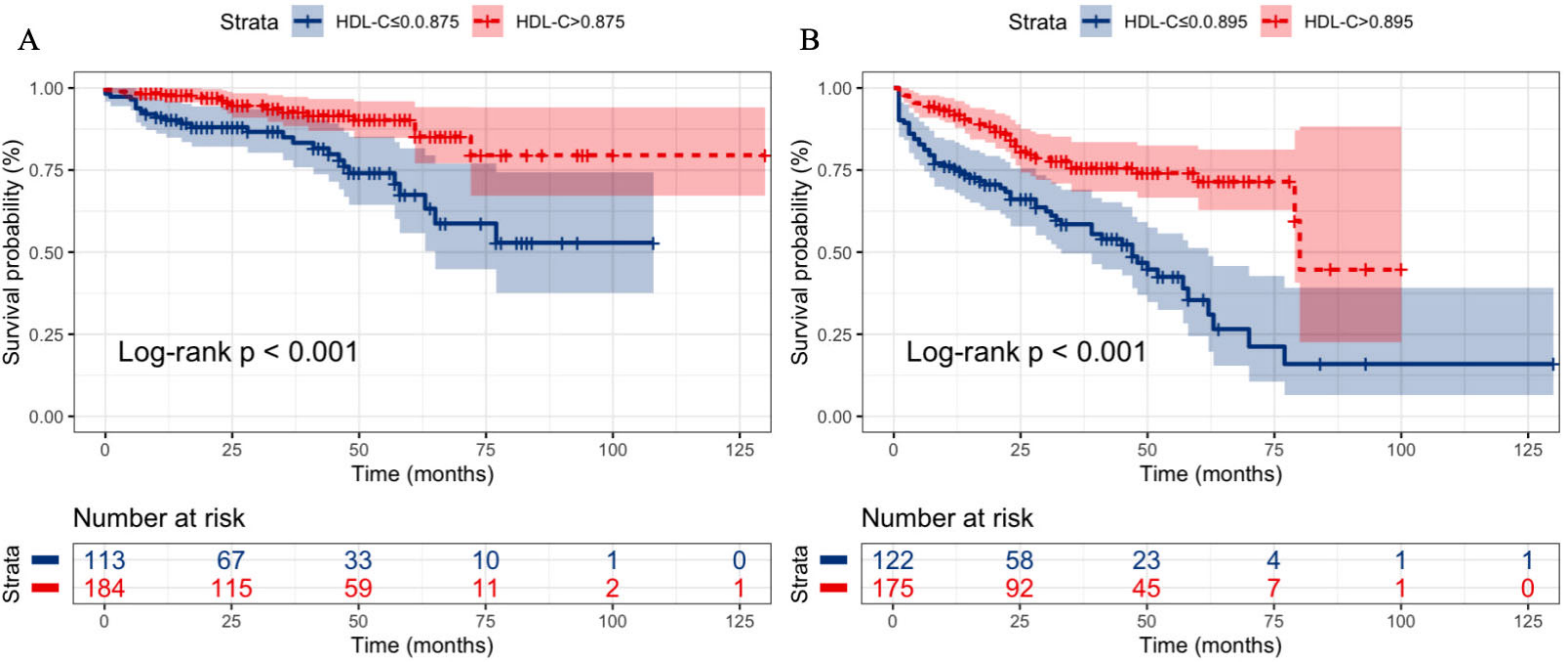
Kaplan-Meier curves for primary **(A)** and secondary **(B)** endpoints stratified by optimal HDL-C cut-offs (Youden method: primary 0.875, secondary 0.895). HDL-C, high-density lipoprotein cholesterol.

ROC analysis identified optimal HDL-C cut-off values of 0.875 for the primary endpoint (Area under the curve [AUC] = 0.621) and 0.895 for the secondary endpoint (AUC = 0.652) (both P < 0.05). The Kaplan-Meier curves for primary and secondary endpoint-free survival are shown in **Figure 1**. Patients with higher HDL-C levels had significantly better event-free survival for both primary and secondary endpoints than those with lower HDL-C levels (log-rank P < 0.001 for both).

**Table 2** presents the Cox regression analyses for the association between HDL-C levels and clinical endpoints. In the unadjusted model (Model 1), higher HDL-C was significantly associated with a reduced risk of the primary endpoint (HR 0.27, 95% CI: 0.09–0.85, P = 0.025) and the secondary endpoint (HR 0.24, 95% CI: 0.12–0.48, P < 0.001). Model 2 adjusted for demographic, clinical, laboratory, and imaging variables, including age, sex, BMI, NYHA class, standard heart failure medications (ARNI/ACEI/ARB, β-blockers, and MRAs), log NT-proBNP, and LVEF. In this model, the associations remained significant for both endpoints (primary: HR 0.19, 95% CI: 0.05–0.68, P = 0.011; secondary: HR 0.15, 95% CI: 0.07–0.34, P < 0.001). In Model 3, which further adjusted for ePASP, HDL-C showed a borderline significant association with the primary endpoint (HR 0.31, 95% CI: 0.08–1.11, P = 0.072). However, it remained significantly and inversely associated with the secondary endpoint (HR 0.19, 95% CI: 0.08–0.44, P < 0.001).

**Table 2:** Association of HDL-C Levels with primary and secondary endpoints.

Models	Primary endpoint			Secondary endpoint		
	HR	95% CI	P-value	HR	95% CI	P-value
Model 1	0.27	0.09, 0.85	0.025	0.24	0.12, 0.48	<0.001
Model 2	0.19	0.05, 0.68	0.011	0.15	0.07, 0.34	<0.001
Model 3	0.31	0.08, 1.11	0.072	0.19	0.08, 0.44	<0.001

Model 1: Unadjusted.

Model 2: Adjusted for age, sex, BMI, NYHA class, standard heart failure medications (ARNI/ACEI/ARB, β-blockers, MRAs), log NT-proBNP, and LVEF.

Model 3: Additionally adjusted for ePASP.

HR, hazard ratios; CI, confidence interval; other abbreviations as in **Table 1**.

**Figure 2:**
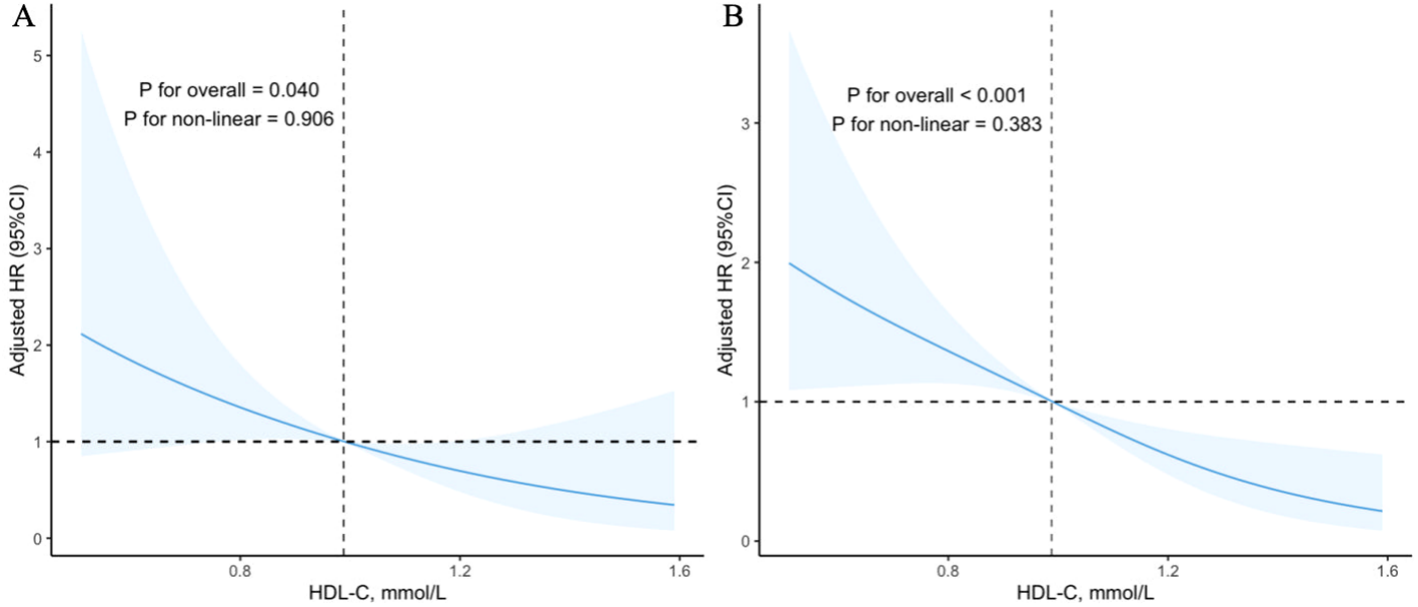
Dose–response relationships between HDL-C and the primary **(A)** and secondary **(B)** endpoints. Hazard ratios (solid lines) and 95% confidence intervals (shaded areas) were adjusted for age, sex, BMI, NYHA class, standard heart failure medications (ARNI/ACEI/ARB, β-blockers, MRAs), log NT-proBNP, and LVEF. Abbreviations are as in **Table 1**.

Using RCS functions with three knots based on Model 2, we further estimated the dose-response relationship between HDL-C and the clinical endpoints. A negative linear association was observed for both the primary (**Figure 2A**) and secondary (**Figure 2B**) endpoints (P for nonlinearity = 0.906 and 0.383, respectively).

### 3.3 Sensitivity analysis and association of HDL-C with PH

The results were generally robust in sensitivity analyses (**Table 3**). HDL-C remained a significant independent risk factor for both endpoints after applying Firth’s penalized likelihood Cox regression or after further adjustment for the inflammatory marker leukocyte count and the nutritional marker albumin (all P < 0.05). Furthermore, when ePASP was replaced with TR velocity as the adjustment variable for PH, a similar attenuation of the effect estimate for the association between HDL−C and clinical outcomes was observed (all P < 0.05).

**Table 3:** Sensitivity analysis of HDL-C associations with clinical endpoints in patients with DCM.

Sensitivity analyses	Primary endpoint			Secondary endpoint		
	HR	95% CI	P-value	HR	95% CI	P-value
Firth’s penalized likelihood Cox regression	0.19	0.06, 0.58	0.003	0.18	0.08, 0.37	<0.001
Additionally adjusted for leukocyte count and albumin	0.18	0.05, 0.70	0.014	0.14	0.06, 0.33	<0.001
Additionally adjusted for tricuspid regurgitation velocity	0.24	0.07, 0.87	0.030	0.18	0.08, 0.41	<0.001

HR, hazard ratios; CI, confidence interval; other abbreviations as in **Table 1**. Each analysis was adjusted for age, sex, BMI, NYHA class, standard heart failure medications (ARNI/ACEI/ARB, β-blockers, MRAs), log NT-proBNP, and LVEF.

**Figure 3:**
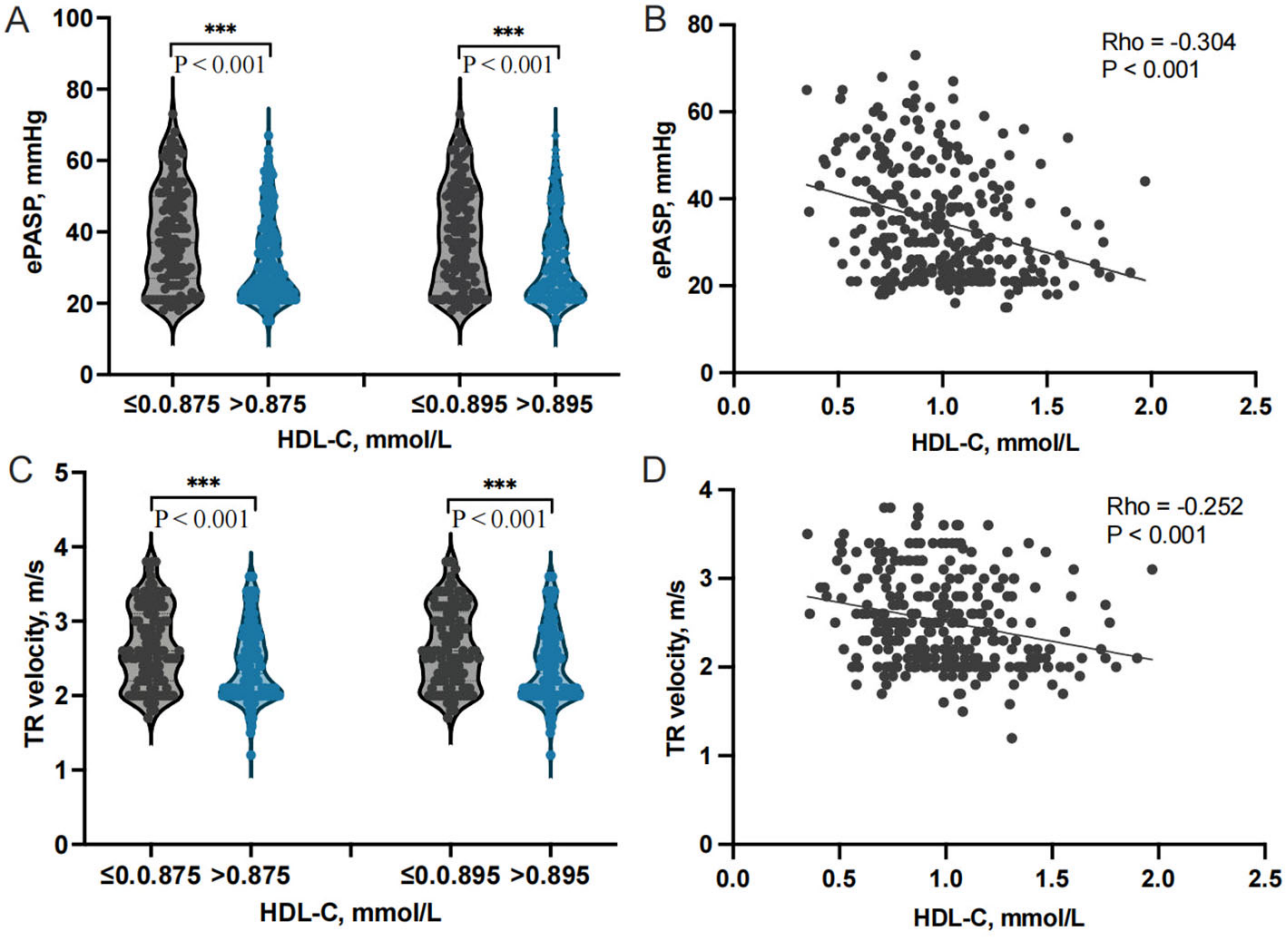
Relationships between HDL-C levels and markers of pulmonary hypertension. **(A, C)** Violin plots displaying the distribution of echocardiographic ePASP and TR velocity categorized by HDL-C groups; **(B, D)** scatter plots of these associations. HDL-C, high-density lipoprotein cholesterol; TR, tricuspid regurgitation; ePASP, estimated pulmonary artery systolic pressure. *** indicates P < 0.001.

**Figure 3** illustrates the relationships between HDL-C levels and echocardiographic markers of PH, including ePASP and TR velocity. Violin plots showed that patients with higher HDL-C levels had significantly higher ePASP and TR velocity than those with lower HDL-C levels (all P < 0.001; Panels A and C). Scatter plots revealed statistically significant negative correlations between HDL-C and both ePASP (Rho = −0.304, P < 0.001; Panel B) and TR velocity (Rho = −0.252, P < 0.001; Panel D), indicating moderate correlation strength.

## 4 Discussion

In this retrospective cohort study of patients with non-ischemic DCM, lower HDL-C levels were significantly associated with an increased risk of adverse clinical outcomes, including all-cause mortality, heart transplantation, and heart failure readmission. This association remained robust after multivariable adjustment but was attenuated upon further adjustment for echocardiographic markers of PH (ePASP and TR velocity), suggesting that PH may partially explain the link between HDL-C and adverse prognosis.

Previous prognostic studies on HDL-C have primarily focused on populations with coronary artery disease and cardiomyopathy, consistently demonstrating that low HDL-C is associated with an increased risk of adverse cardiovascular events (17, 18). For example, Yang et al. conducted a prospective study in patients with light-chain cardiac amyloidosis and found that lower serum HDL-C levels were associated with more severe cardiac injury and shorter survival, with HDL-C level confirmed as an independent prognostic factor (19). In addition, Kaur et al. reported a U-shaped relationship between HDL-C and all-cause mortality in over 15,000 patients undergoing percutaneous coronary intervention (PCI), with the lowest mortality risk observed at HDL-C levels between 30–50 mg/dL (20). Similarly, Chen et al. found a V-shaped relationship in 7,284 PCI patients, where both low (≤ 25 mg/dL) and high (> 60 mg/dL) HDL-C levels were associated with increased mortality, while moderate levels (25–60 mg/dL) were associated with the most favorable outcomes (21). However, in patients with DCM—particularly those in whom ischemic causes have been rigorously excluded—the prognostic value of HDL-C remains underexplored. Our study fills this gap by systematically evaluating the association between HDL-C levels and long-term clinical outcomes in a well-defined DCM cohort.

Emerging evidence suggests that the cardiovascular benefits of HDL-C extend beyond its role in lipid transport. HDL-C exerts multiple protective effects, including antioxidant, anti-inflammatory, anti-apoptotic, and vasodilatory properties, which are critical for maintaining endothelial integrity and attenuating myocardial injury (5). Recent studies have highlighted that HDL functionality—rather than absolute HDL-C levels alone—may better reflect its biological relevance in cardiovascular diseases. For example, Schrutka et al. assessed the antioxidant function of HDL in 320 patients with chronic heart failure using the HDL oxidative index (HOI) (22). They found that an elevated HOI, indicative of impaired HDL antioxidant capacity, was significantly associated with an increased risk of cardiovascular death and heart transplantation. Similarly, Singh et al. reported that heart transplant patients exhibited impaired cholesterol efflux capacity and diminished vasculoprotective activity of HDL, despite normal HDL-C levels (23). Patel et al. also demonstrated reduced antioxidant capacity and efflux function of HDL in patients with ischemic cardiomyopathy (24). At the cellular level, Wu et al. showed that HDL pretreatment in cardiomyoblasts attenuated palmitic acid-induced lipotoxicity, reduced reactive oxygen species accumulation, and stabilized mitochondrial function, thereby suppressing apoptosis (25). Although the present study did not directly assess HDL functionality, these mechanistic insights provide a biological rationale for the potential link between HDL-C and pulmonary vascular dysfunction.

In the context of our study, we observed that lower HDL-C levels were associated with elevated echocardiographic markers of PH, including TR velocity and ePASP. Furthermore, adjustment for PH attenuated the association between HDL-C and adverse outcomes. This descriptive finding suggests that PH may partly explain the observed association, which is consistent with the hypothesis that impaired HDL function could contribute to pulmonary vascular remodeling and increased right ventricular afterload—factors known to influence prognosis in DCM patients. However, given the cross-sectional measurement of HDL-C and PH variables, we emphasize that this interpretation is exploratory and does not establish causality. Future studies incorporating invasive hemodynamic assessment and direct measures of HDL functionality are needed to further elucidate these relationships.

This study has several limitations. First, it was a single-center retrospective study with a relatively small sample size and a limited number of events. This may introduce selection bias and limit the generalizability of the findings. Second, although we simplified the multivariable model to maintain an acceptable events-to-covariates ratio (approximately 5:1), residual confounding and potential overfitting cannot be fully excluded. Notably, important confounders—including sodium-glucose cotransporter 2 inhibitors (SGLT2i) use, statin use, and cardiac fibrosis assessed by CMR—were not available in our cohort because they were not systematically recorded. Third, PH was assessed using echocardiographic estimates (ePASP derived from TR peak velocity) rather than the gold standard of right heart catheterization. This may introduce measurement error and non-differential misclassification, particularly in patients with significant TR or right ventricular dysfunction. Finally, only baseline HDL-C levels were evaluated; we did not account for dynamic changes over time or functional characteristics of HDL, such as cholesterol efflux capacity or antioxidant function. Future prospective, multicenter studies with larger sample sizes, invasive hemodynamic assessment, and comprehensive confounder adjustment are warranted to validate our findings.

## 5 Conclusions

In summary, lower HDL-C is independently associated with worse prognosis in non-ischemic DCM. Our exploratory analysis suggests that PH may partially account for this relationship. As an easily obtainable lipid parameter, HDL-C holds promise as a pragmatic tool for risk stratification. Future prospective, multicenter studies using invasive hemodynamic assessment via right heart catheterization, along with comprehensive measures of HDL functionality, are needed to further elucidate the underlying mechanisms.

## Data Availability

The raw data supporting the conclusions of this article will be made available by the authors, without undue reservation.
